# 1648. Impact of a Pediatric Antimicrobial Stewardship Program on Optimizing Antibiotic Usage in a Non-Freestanding Children’s Hospital (PEDS-ASP)

**DOI:** 10.1093/ofid/ofad500.1482

**Published:** 2023-11-27

**Authors:** Anita Siu, Linda Barron, Michelle Kohute, Sarah A Rawstron, Amy A Eller, Liliana Cruz, Mariawy Riollano Cruz

**Affiliations:** Rutgers University/Jersey Shore University Medical Center, Piscataway, New Jersey; Jersey Shore University Medical Center, Freehold, New Jersey; Jersey Shore University Medical Center, Freehold, New Jersey; Jersey Shore University Medical Center, Freehold, New Jersey; Jersey Shore University Medical Center, Freehold, New Jersey; Jersey Shore University Medical Center, Freehold, New Jersey; Hackensack Meridian School of Medicine, Neptune, New Jersey

## Abstract

**Background:**

Antibiotic misuse can lead to poor patient outcomes, including ineffective treatment of infections, increased adverse effects, and development of antibiotic resistance. Implementing an antimicrobial stewardship program (ASP) is a vital strategy to improve and measure the appropriate use of antibiotics. This study aimed to assess the impact of pediatric ASP implementation on the reduction of restricted antibiotic usage and the improvement of patient outcomes.

**Methods:**

A single-center retrospective-prospective study was conducted at a children’s hospital and included patients admitted to general pediatric or pediatric intensive care units receiving one or more restricted antibiotics. Prior-authorization of these restricted antibiotics was implemented as the ASP strategy. A three-month pre- and post-implementation chart review was completed. The primary outcome was optimizing restricted antibiotic usage. Secondary outcomes included provider compliance, antibiotic days of therapy (DOT), hospital length of stay (LOS), and readmissions within 30 days.

Table 1

**Figure:**
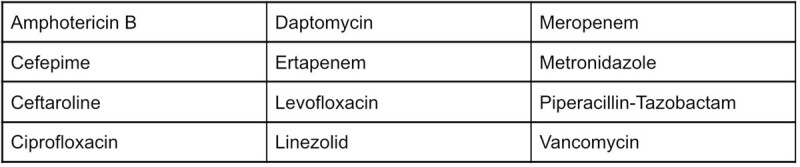

Restricted antibiotics

**Results:**

50 patients were included in the study. Restricted antibiotic orders for 18 out of 24 patients in the post-implementation group (75%) were compliant with obtaining pre-approval. Vancomycin, cefepime, and metronidazole were the most common agents ordered. The most common indications were skin and soft tissue infections, musculoskeletal infections, and preemptive sepsis coverage. Patients receiving restricted antibiotics decreased by two (-7.7%) while total restricted antibiotic orders increased by five (16.7%) following ASP implementation. Mean antibiotic DOT increased by 2.02 days (42.7%) and mean hospital LOS increased by 1.8 days (31.0%) in the post-implementation group. There was no difference in 30-day readmissions.

Figure 1

**Figure:**
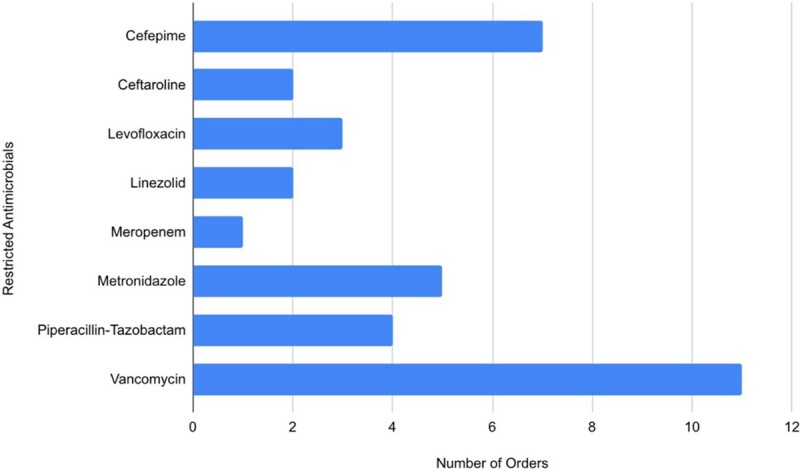

Restricted antibiotics ordered post-implementation of a pediatric antimicrobial stewardship program.

Figure 2

**Figure:**
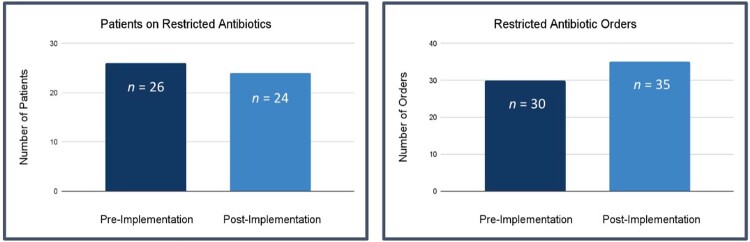

Use of restricted antibiotics pre- and post-implementation of a pediatric antimicrobial stewardship program

**Conclusion:**

The results of this study not only aided in establishing baseline data but also provided a starting point for future efforts concerning the appropriate use of restricted antibiotics. While the findings were inconsistent with those of other institutions, incomplete program compliance and patient complexity in the post-implementation group likely contributed to the increase in DOT and hospital LOS. A longer study duration is needed to assess the true impact of this program.

**Disclosures:**

**All Authors**: No reported disclosures

